# Higher Medical Education

**Published:** 1894-11

**Authors:** 


### *EDITORIAL.*

---

The office of THE JOURNAL is in room 330 Equitable Building.

Address all communications, and make all remittances payable to THE ATLANTA MEDICAL AND SURGICAL JOURNAL, Atlanta, Ga.

Articles for publication should reach this office not later than the 15th instant.

The editors are not responsible for views expressed by contributors.

---

### HIGHER MEDICAL EDUCATION.

We publish in another place an excellent paper on this subject by Dr. Elkin, of this city. We may take this occasion to add a few words of our own, which will have a special appropriateness at this time.

We make no apology for the prominence which this JOURNAL has given to the need for better medical education and proper medical legislation. No subjects can be more important than these, either to us as physicians or to this JOURNAL as an exponent of medical thought and progress. We have labored faithfully along these lines. This has been the *Carthago delenda est* which we have sounded in the ears of our readers almost every month for three years. And the end is not yet.

What we wish to say now will have reference mainly to the establishment of new colleges in their relation with higher medical education. And right here we take our stand firmly upon the statement of Dr. Elkin, that what we need in the South is not more colleges, but fewer colleges, with thoroughly equipped laboratories and better clinical advantages. This has been our position from the first.

A new medical college was organized the other day over in Alabama. Also a new one at Fort Worth, Texas. About one month ago two members of the faculty of one of the Nashville schools resigned their chairs on account of personal differences, and are now industriously at work "organizing another school." More schools than this have been established from no better motive. Out in St. Louis the opening of a new medical college has gotten to be almost a joke. In a word, there is absolutely *no need for these colleges*. There is every need, God knows, for better medical education, but the way to accomplish it is not by increasing the number of colleges. Since 1880 there have been more than fifty medical schools chartered in this country, a great many more than there was any necessity for. Fortunately several have died. There are 110,000 doctors in the United States. More than 3,000 belong to the faculties of medical schools. Of this number Ohio, with 16 schools, contributes 408; New York, with 12 schools, 540; Missouri, with 16 schools, 405; Maryland, with 7 schools, 170; and Tennessee, with 8 schools, 160, etc. In all we have one medical school for every 430,000 of our population, or one for every 730 physicians.

What wonder is it that the American diploma has such a poor rating abroad. And the work of making new schools still goes on. Dr. Jerome Cochran, of Alabama, has lately said: "We have already in this country three times—yes, six times—as many medical colleges as we have any need for. In this opinion I will be sustained by nine-tenths of the doctors in the United States. This being so, the further multiplication of medical colleges is not a consummation devoutly to be wished; and a new medical college in Alabama can by no possibility be of any benefit to anybody except the gentlemen personally connected with it. It does not fill a long-felt want. . . . The process of creating a new college is simple enough. It is only necessary to form a company of seven or eight doctors, build a big brick house, and apply to a complaisant legislature for a charter, and lo! the thing is done."

We know that public sentiment in the profession is against this rapid proliferation of medical colleges, in the South and elsewhere. One-third of our present number of colleges would be amply sufficient, and would enable those engaged in medical teaching to throw in a medical education along with every diploma. Too many of our colleges have as their only excuse for living the "private emolument, either in money or reputation, of the men who built them." Too many of them are what Mr. Cleveland would call a "Communism of Pelf." Such being the case, and if colleges continue to multiply out of all proportion to the public demand for them, then the proper standard of medical education will ever remain an "iridescent dream."

We have already in this country vastly too many doctors, the ill-begotten and premature products of the colleges. In their exercise of the diploma-conferring power, the colleges owe a certain responsibility both to the public and the profession. This responsibility will not be met by institutions organized and conducted on the basis of a college for revenue only. There are more than enough colleges in this country right now to satisfy every demand for twenty years to come. But others will be established, more or less rapidly, and their multiplication will have only one effect—to hinder and retard, what some conscientious teachers hope to witness some day, a high and uniform standard of medical education, which will be a pride and a delight to the American medical profession.

